# Gene Expression Profiles for Macrophage in Tissues in Response to Different Exercise Training Protocols in Senescence Mice

**DOI:** 10.3389/fspor.2019.00050

**Published:** 2019-10-18

**Authors:** Masataka Uchida, Naoki Horii, Natsuki Hasegawa, Shumpei Fujie, Eri Oyanagi, Hiromi Yano, Motoyuki Iemitsu

**Affiliations:** ^1^Faculty of Sport and Health Science, Ritsumeikan University, Kusatsu, Japan; ^2^Japan Society for the Promotion of Science, Tokyo, Japan; ^3^Research Organization of Science and Technology, Ritsumeikan University, Kusatsu, Japan; ^4^Faculty of Health and Sciences, University of Tsukuba, Tsukuba, Japan; ^5^Department of Health and Sports Science, Kawasaki University of Medical Welfare, Kurashiki, Japan

**Keywords:** aging, macrophage polarization, chemokine, exercise training, chronic inflammation

## Abstract

Age-induced chronic inflammation is prevented by aerobic and resistance exercise training. However, the effects of the mechanism of exercise on chronic inflammation in each tissue remains unclear. The aim of this study was to investigate the effects of resistance and aerobic training on gene expression profiles for macrophage infiltration and polarization (M1/M2 ratio) with chronic inflammation in various tissues of aged model mice. Male 38-week-old SAMP1 (senescence-accelerated prone mouse 1) mice were randomly divided into three groups—sedentary (Aged-Sed-SAMP1), aerobic training (Aged-AT-SAMP1; voluntary running), and resistance training—for 12 weeks (Aged-RT-SAMP1; climbing ladder). Resistance and aerobic exercise training prevented an increase in circulating TNF-α levels (a marker of systemic inflammation) in aged SAMP1 mice, along with decreases in tissue inflammatory cytokine (TNF-α and IL-1β) mRNA expression in the heart, liver, small intestine, brain, aorta, adipose, and skeletal muscle, but it did not change the levels in the lung, spleen, and large intestine. Moreover, resistance and aerobic exercise training attenuated increases in F4/80 mRNA expression (macrophage infiltration), the ratio of CD11c/CD163 mRNA expression (M1/M2 macrophage polarization), and MCP-1 mRNA expression (chemokine: a regulator of chronic inflammation) in the chronic inflamed tissues of aged SAMP1 mice. These results suggested that resistance and aerobic exercise training-induced changes in gene expression for macrophage infiltration and polarization in various tissues might be involved in the prevention of age-related tissue chronic inflammation, and lead to a reduction of the increase in circulating TNF-α levels, as a marker of systemic inflammation, in aged SAMP1 mice.

## Introduction

Aging induces the dysregulation of immune function and leads to systemic inflammation (Woods et al., 2012). At both circulating and tissue levels, the levels of pro-inflammatory cytokines, such as tumor necrosis factor (TNF)-α, interleukin-6 (IL-6), and interleukin-1β (IL-1β), increase with an increase in age in both humans and/or mice (Brüünsgaard and Pedersen, 2003; Franceschi, 2007; Freund et al., 2010; Woods et al., 2012). Age-induced chronic inflammation in various tissues may be largely attributed to age-related diseases, such as metabolic disorders, Alzheimer's disease, cancer, bowel diseases, sarcopenia, and cardio vascular diseases (Hotamisligil et al., 1993; Weiner and Selkoe, 2002; Brüünsgaard and Pedersen, 2003; Hansson et al., 2006; Libby, 2007; Tilg et al., 2008; Freund et al., 2010; Woods et al., 2012). The tissues inflamed by aging show an infiltration of macrophages (Mosser and Edwards, 2008). The macrophage infiltration in the tissue is accelerated by the upregulation of the expression of chemokines, such as macrophage chemotactic protein-1 (MCP-1), which are secreted from accumulated senescent cells by a downregulation of macrophage phagocytotic activity (Oishi and Manabe, 2016). Macrophage activation has been classified according to two polarized activation states, namely pro-inflammatory M1 (specifically express CD11c and CCR2) and anti-inflammatory M2 (specifically express CD163) macrophages in non-pathogen-driven conditions (Varin and Gordon, 2003; Sica and Mantovani, 2012). Therefore, since M1 macrophages contribute to induced chronic inflammation and M2 macrophages inhibit these inflammatory reactions (Lee et al., 2013; Dimitrijević et al., 2016), macrophage infiltration and the balance of macrophage polarization are key regulators for age-induced chronic inflammation in various tissues.

Regular aerobic exercise reduces the circulating levels of inflammatory markers, such as TNF-α and IL-6, in aged mice and humans (Packer and Hoffman-Goetz, 2012; Santos et al., 2012; Woods et al., 2012). In animal studies, the enhancement of chronic inflammation with macrophage infiltration due to aging or obesity in the aortic or liver tissues only is attenuated by aerobic exercise training (Lesniewski et al., 2011; Kawanishi et al., 2013). Additionally, resistance training also reduces the circulating levels of inflammatory markers (plasma TNF-α concentration) in older adults; however, the effect on tissue levels is unknown (Chupel et al., 2017). Furthermore, the effect of chronic exercise training on chronic inflammation may differ between aerobic and resistance training. However, the preventive effects of different exercise types (such as aerobic and resistance training) on age-induced chronic inflammation in each tissue remains unclear. Moreover, a mechanism underlying the preventive effect of aerobic and resistance training on chronic inflammation remains unclear.

In this study, we aimed to elucidate whether resistance and aerobic exercise training altered the gene expression profiles for macrophages in various tissues of aged mice, which could potentially result in the attenuation of age-related chronic inflammation. We hypothesized that the effect of continuous exercise training on chronic inflammation-related gene profiles may differ between aerobic and resistance training. To test the specificity of different exercise training on age-related macrophage profiles in various tissues, we investigated the effects of resistance and aerobic training on gene expression for macrophage infiltration as well as polarization (from M1 to M2) with chronic inflammatory responses in various tissues of senescence-accelerated prone mouse 1 (SAMP1) mice, a model of aging mice. SAMP1 mice exhibit accelerated senescence and early occurrence of age-related chronic diseases, such as chronic inflammation of the arterial wall and adipose tissue, dysregulated immune function, senile amyloidosis, sarcopenia, and degenerative joint disease, without any experimental manipulation (Takeda et al., 1991; Haramizu et al., 2011; Chen et al., 2018; Horii et al., 2018). These pathological characteristics observed in SAMP1 mice are similar to those observed in a spontaneously aging standardized mouse model (i.e., C57BL/6 mice).

## Materials and Methods

### Animals and Protocol

Ethical approval for this study was obtained from the Committee on Animal Care at Ritsumeikan University. Male senescence-accelerated prone mouse 1 (SAMP1) mice (8 weeks old) were obtained from Japan SLC (Shizuoka, Japan) and cared for according to the Guiding Principles for the Care and Use of Animals, based on the Declaration of Helsinki. The mice were housed individually in cages under controlled conditions on a 12:12 h light-dark cycle and were allowed *ad libitum* access to food (MF; CLEA Japan, Tokyo, Japan) and water for the duration of the experimental period. Ten 13-week-old SAMP1 mice were used as a young sedentary (Young-Sed -SAMP1), and thirty 38-week-old SAMP1 mice were randomly divided into three groups: aged sedentary (Aged-Sed-SAMP1, *n* = 10), aged aerobic training (Aged-AT-SAMP1, *n* = 10), and aged resistance training (Aged-RT-SAMP1, *n* = 10).

Post-treatment experiments on Aged-AT-SAMP1 and Aged-RT-SAMP1 mice were performed more than 48 h after the last exercise session to avoid the acute effects of each training exercise intervention. Additionally, chow was removed from the cages of all mice for 12 h, and after measuring the body weight, the blood samples were obtained from the orbital eye vessel under general anesthesia. The blood samples were collected in EDTA-coated tubes and immediately placed on ice. Plasma was isolated from whole blood by centrifugation (2,000 × g, 20 min at 4°C) and stored at −80°C until other assays were performed. In addition, the brain, heart, lung, liver, kidney, spleen, aorta, epididymal fat, small intestine, large intestine, quadriceps femoris muscle, and tibialis anterior (TA) muscle samples were resected quickly, rinsed in ice-cold saline, frozen in liquid nitrogen, and stored at −80°C for further analysis.

### Exercise Training Protocol

As indicated in previous studies, the Aged-RT-SAMP1 group completed resistance training three times a week for 12 weeks using a climbing ladder (90 cm, 1 cm grid, 80° incline) (Matheny et al., 2009; Horii et al., 2018). For the training intervention, 50% of body weight was attached to the tails of the mice, and the weight was progressively increased to 75, 90, and 100% of individual body weight. If the mice could climb with 100% of their body weight, the weight was progressively increased by 3.0 g. The trained mice climbed the ladder with a load that allowed them to perform 6–8 sets of the exercise; the mice were allowed to rest for 1 min between sets. The training was conducted between 8:00 and 9:00 p.m. during the dark phase.

As indicated in previous studies, the Aged-AT-SAMP1 group exercised by voluntary running on a wheel (diameter 15.5 cm, Brain science idea, Osaka, Japan) (Littlefield et al., 2015). Aged-AT-SAMP1 mice were housed in cages with free access to a running wheel for 12 weeks. The running distance was electronically monitored and recorded daily by a magnetic switch interfaced to a computer using Wheel Manager Data Acquisition Software.

### Measurement of Citrate Synthase (CS) Activity

Quadriceps femoris muscle tissues (20 mg) were homogenized in 10 volumes of 250 mM sucrose, 1 mM Tris·HCl (pH 7.4), and 130 mM NaCl on ice using a Teflon homogenizer. The homogenate was centrifuged at 9,000 g for 20 min at 0°C, and the pellet was resuspended in homogenate buffer and centrifuged at 600 g for 10 min at 0°C. The resultant supernatant was centrifuged at 8,000 g for 15 min at 0°C, and the pellet was resuspended in 250 mM sucrose. To determine the citrate synthase activity, 8 μl of each sample was incubated for 2 min at 30°C in a 182-μl incubation mixture containing 100 mM Tris·HCl (pH 8.0), 1 mM 5,5-dithiobis [2-nitrobenzoic acid], and 10 mM acetyl-CoA. The reaction was initiated by the addition of 10 μl of 10 mM oxaloacetate and was then determined spectrophotometrically at 412 nm for 3 min.

### Measurement of TNF-α

Plasma TNF-α (R&D Systems, Minneapolis, MN, USA) concentration was determined using a high sensitivity TNF-α sandwich enzyme-linked immunosorbent assay (ELISA) kit. The absorbance was measured at 450 nm by microplate reader using an xMark microplate spectrophotometer (Bio-Rad Laboratories, Hercules, CA, USA).

### Real-Time RT-PCR

Total tissue RNA in brain, lung, heart, liver, kidney, spleen, small intestine, large intestine, aorta, and TA tissues was isolated using the ISOGEN reagent (Nippon Gene, Toyama, Japan) and the RNeasy mini kit (QIAGEN, Hilden, Germany), as previously described (Matheny et al., 2009; Horii et al., 2018). Single-stranded cDNA was synthesized from prepared RNA (2 μg) using OmniScript reverse transcriptase (QIAGEN, Hilden, Germany). The mRNA expression of inflammatory cytokines (IL-6, IL-1β and TNF-α,), chemokine (monocyte chemotactic protein-1: MCP-1), and macrophage markers (F4/80, CD11c, and CD163) in these tissues were analyzed by real-time PCR with TaqMan Gene Expression assays (*IL-6* was identified as Mm00446190_m1, *IL-1*β as Mm00434228_m1, *TNF-a* as Mm00441242_m1, *F4/80* as Mm00802529_m1, *CD11c* as Mm00498701_m1, *CD163* as Mm0047091_m1, *CCR2* as Mm_99999051_gH, *MCP-1* as Mm00441242_m1; Applied Biosystems, Foster City, CA, USA), as previously described (Macpherson et al., 2015; Horii et al., 2018). Real-time PCR was performed on a Prism 7500 Fast Sequence Detection System 2.2 (Applied Biosystems), and cycle threshold values were calculated using the system software. These mRNA expression levels were normalized against the expression levels of GAPDH mRNA in the same sample (assay ID, Mm99999915_g1; Applied Biosystems).

### Statics Analysis

All values are expressed as the mean ± SEM. Statistical evaluations were performed using one-way ANOVA. The Fisher *post-hoc* test was used to correct for multiple comparisons when analyses revealed significant differences. Data were considered to be significantly different when *p*-values were less than 0.05.

## Results

### Animal Characteristics

Thirty-eight-week-old senescence accelerated prone mouse 1 (SAMP1) mice carried out aerobic training (Aged-AT-SAMP1 group), resistance training (Aged-RT-SAMP1 group), or remained sedentary (Aged-Sed-SAMP1 group) for 12 weeks. Additionally, 25-week-old SAMP1 mice were used as a young sedentary (Young-Sed-SAMP1 group). Body weight was significantly lower in the Aged-RT-SAMP1 and Aged-AT-SAMP1 groups than in the Aged-Sed-SAMP1 group but did not change compared with that in the Young-Sed-SAMP1 group (Table 1). LV mass/body weight was significantly increased in the Aged-AT-SAMP1 group compared to that in the Aged-Sed-SAMP1 group (Table 1). Adipose tissue mass was decreased in the Aged-AT-SAMP1 group compared to that in the Aged-Sed-SAMP1 group (Table 1). Additionally, in the Aged-Sed-SAMP1 group, TA muscle mass was lower than that in the Young-Sed-SAMP1 group (*p* = 0.0528), whereas the Aged-RT-SAMP1 group showed a significant increase in TA muscle mass when compared with the Aged-Sed-SAMP1 and the Aged-AT-SAMP1 groups (Table 1). Muscle CS activity in the Aged-Sed-SAMP1 group significantly decreased compared to that in the Young-Sed-SAMP1 group, whereas the Aged-AT-SAMP1 group exhibited a significant increase in muscle CS activity compared with the Aged-Sed-SAMP1 and the Aged-RT-SAMP1 groups (Table 1). Furthermore, the muscle CS activity in the Aged-RT-SAMP1 group was higher than in the Aged-Sed-SAMP1 group (Table 1, *p* = 0.0503). In the aged SAMP1 mice, the food intake of resistance training mice was not significantly different when compared to that of sedentary mice.

**Table 1:** Animal characteristics.

	**Young-Sed-SAMP1 (*n* = 10)**	**Aged-Sed-SAMP1 (*n* = 10)**	**Aged-AT-SAMP1 (*n* = 10)**	**Aged-RT-SAMP1 (*n* = 10)**
Body weight (g)	35.3 ± 1.1	36.4 ± 1.2	36.0 ± 3.7^†^	33.9 ± 1.5^††^
LV mass (mg)	177.9 ± 13.1	196.2 ± 11.3	213.3 ± 6.2	182.7 ± 6.2
LV mass/body weight (mg/g)	5.0 ± 0.3	5.4 ± 0.3	6.4 ± 0.3^†^	5.7 ± 0.2
Adipose tissue mass (g)	1.7 ± 0.1	1.4 ± 0.2	1.0 ± 0.2^†^	1.2 ± 0.2
Tibialis anterior muscle mass (mg)	53.8 ± 0.7	49.2 ± 1.5	51.7 ± 1.6	58.3 ± 1.1^††^^#^
TA muscle mass/body weight (mg/g)	1.5 ± 0.04	1.4 ± 0.06	1.5 ± 0.08	1.8 ± 0.04^††^^#^
Muscle citrate synthase activity _(mol/min/gtissue)_	13.2 ± 0.8	11.1 ± 0.7^*^	15.2 ± 0.4^††^	13.0 ± 0.7^#^
Food intake (g/day)	3.5 ± 0.1	3.2 ± 0.1	4.2 ± 0.5^††^	3.2 ± 0.1^#^

*Values are means ± SEM. Young-Sed-SAMP1, young sedentary group; Aged-Sed-SAMP1, aged sedentary group; Aged-AT-SAMP1, aged aerobic training group; Aged-RT-SAMP1; aged resistance training group. LV, left ventricular*.

* p < 0.05 vs.Young-Sed;

† *p < 0.05 vs. Aged-Sed-SAMP1*;

†† *p < 0.01 vs. Aged-Sed-SAMP1*;

# *p < 0.05 vs. Aged-AT-SAMP1*.

### Plasma TNF-α Concentration

**Figure 1:**
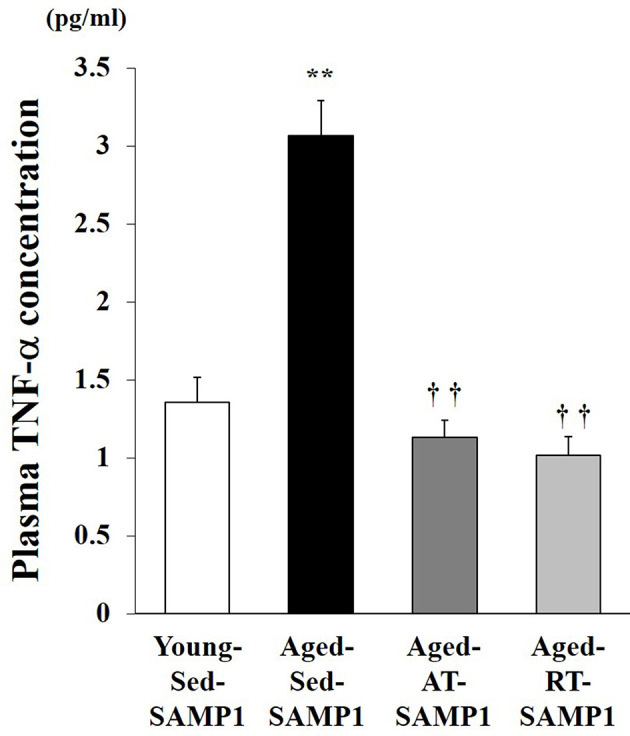
Effects of aging and exercise training on plasma TNF-α level. Young-Sed-SAMP1, young sedentary group; Aged-Sed-SAMP1, aged sedentary group; Aged-AT-SAMP1, aged aerobic training group; Aged-RT-SAMP1, aged resistance training group. The values are expressed as means ± SEM. ^**^p < 0.01 vs. Young- Sed-SAMP1; ^††^*p* < 0.01 vs. Aged- Sed-SAMP1.

This study examined plasma TNF-α levels, which are a marker of systemic inflammation, in young and aged SAMP1 mice. Plasma TNF-α levels significantly increased in the Aged-Sed-SAMP1 group compared to those in the Young-Sed-SAMP1 group (Figure 1). However, plasma TNF-α levels in the Aged-RT-SAMP1 and the Aged-AT-SAMP1 groups significantly decreased compared to those in the Aged-Sed-SAMP1 group (Figure 1).

### Tissue Inflammatory Cytokines mRNA Expression

**Figure 2:**
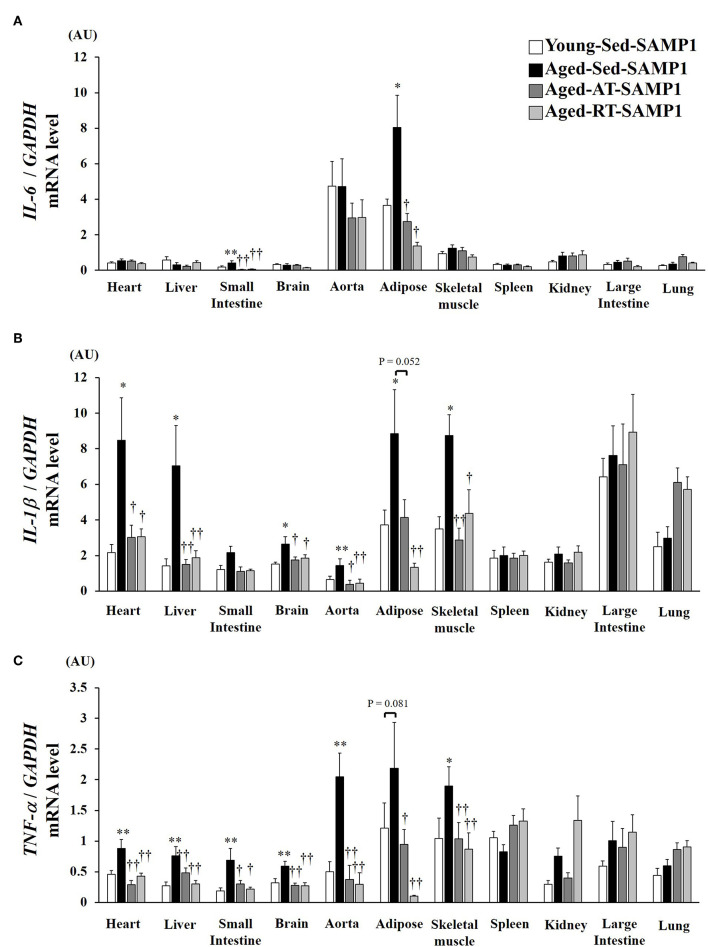
Effect of aging and exercise training on *IL-6* **(A)**, *IL-1*β **(B)**, and *TNF-*α **(C)** mRNA expression in tissues. *GAPDH* mRNA expression was used as an internal control for correcting the mRNA expression of *IL-6, IL-1*β, and *TNF-*α in the heart, liver, small intestine, brain, aorta, adipose, skeletal muscle (tibialis anterior muscle), spleen, kidney, large intestine, and lung tissues. AU, arbitrary units. The values are expressed as means ± SEM. ^*^p < 0.05 vs. Young- Sed-SAMP1; ^**^p < 0.01 vs. Young- Sed-SAMP1; ^†^*p* < 0.05 vs. Aged- Sed-SAMP1; ^††^*p* < 0.01 vs. Aged- Sed-SAMP1.

To confirm the chronic inflammatory state in various tissues, we assessed mRNA expressions of inflammatory cytokines in various tissues. IL-6 mRNA expression in the adipose and the small intestine significantly increased in the Aged-Sed-SAMP1 group compared to that in the Young-Sed-SAMP1 group (Figure 2A). However, IL-6 mRNA expression in the Aged-AT-SAMP1 and the Aged-RT-SAMP1 groups was reduced compared to that in the Aged-Sed-SAMP1 group (Figure 2A). No significant difference in IL-6 mRNA expression was observed across the four groups in the other tissues (Figure 2A). Additionally, IL-1β mRNA expression in the heart, liver, small intestine, aorta, adipose, and TA muscle tissues significantly increased in the Aged-Sed-SAMP1 group compared to that in the Young-Sed-SAMP1 group (Figure 2B). However, IL-1β mRNA expression in the heart, liver, small intestine, aorta, and TA muscle was reduced by resistance and aerobic training (Figure 2B). Moreover, IL-1β mRNA expression in adipose tissue was significantly lower in the Aged-RT-SAMP1 and the Aged-AT-SAMP1 groups than in the Aged-Sed-SAMP1 group (Figure 2B, *p* = 0.003, *p* = 0.052, respectively). Additionally, TNF-α mRNA expression in the heart, liver, small intestine, brain, aorta, and TA muscle significantly increased in the Aged-Sed-SAMP1 group compared to that in the Young-Sed-SAMP1 group (Figure 2C). TNF-α mRNA expression in the adipose tissue of the Aged-Sed-SAMP1 group tended to be higher than in the Young-Sed-SAMP1 group (Figure 2C). However, the TNF-α mRNA expression levels in the Aged-AT-SAMP1 and the Aged-RT-SAMP1 groups were reduced compared to those in the Aged-Sed-SAMP1 group, but the levels were not reduced compared to those in the Young-Sed-SAMP1 group (Figure 2C). No significant difference in TNF-α mRNA expression in the spleen, large intestine, and lung was found in the Aged-Sed-SAMP1 group compared with the Young-Sed-SAMP1 group (Figure 2C).

### Infiltration and Polarization of Macrophages

**Figure 3:**
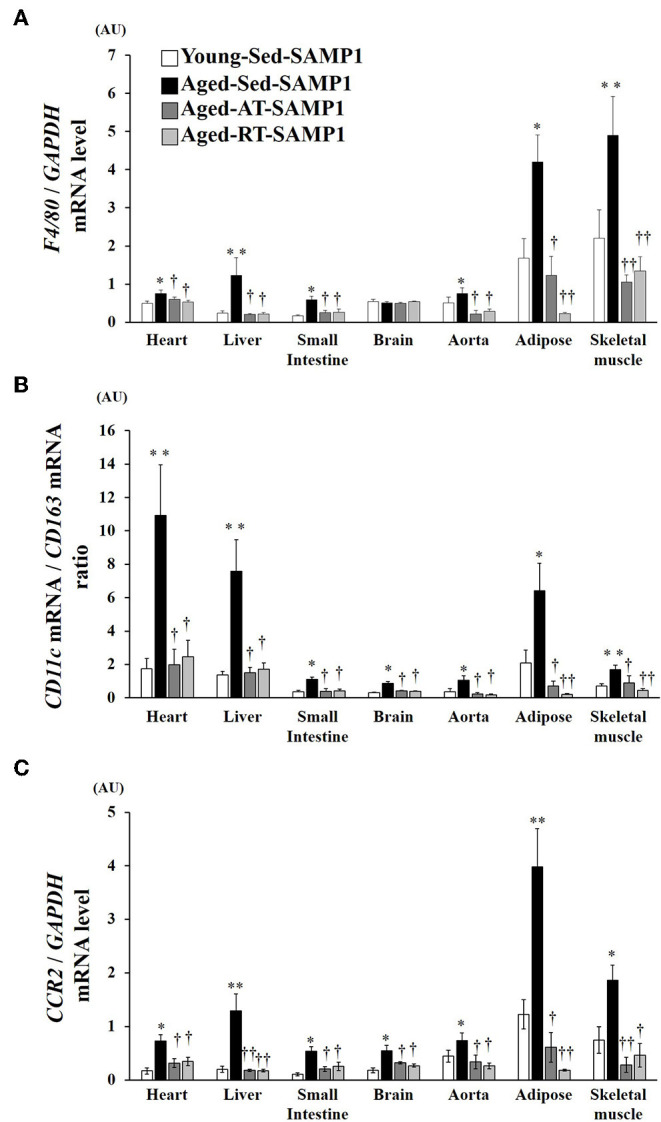
Effects of aging and exercise training on *F4/80* mRNA expression **(A)**, ratio of *CD11c*/*CD163* mRNA expression **(B)** and *CCR2* mRNA expression **(C)** in inflamed tissues. *GAPDH* mRNA expression was used as an internal control for correcting the mRNA expression of *F4/80, CD11c, CD163*, and *CCR2* in the heart, liver, small intestine, brain, aorta, adipose, and skeletal muscle (tibialis anterior muscle) tissues. Ratio of *CD11c*/*CD163* mRNA expression in the heart, liver, small intestine, brain, aorta, adipose, and skeletal muscle (tibialis anterior muscle) tissues is shown. AU, arbitrary units. The values are expressed as means ± SEM. ^*^p < 0.05 vs. Young- Sed-SAMP1; ^**^p < 0.01 vs. Young-Sed-SAMP1; ^†^*p* < 0.05 vs. Aged-Sed-SAMP1; ^††^*p* < 0.01 vs. Aged-Sed-SAMP1.

To examine macrophage infiltration and polarization, we analyzed the expression of macrophage markers by mRNA in inflamed tissues. In the heart, liver, small intestine, aorta, adipose, and TA muscle samples of the Aged-Sed-SAMP1 group, F4/80 mRNA expression was significantly higher than that in the Young-Sed-SAMP1 group but was not altered in the brain (Figure 3A). In contrast, F4/80 mRNA expression significantly decreased in the heart, liver, small intestine, aorta, adipose, and TA muscle of the Aged-RT-SAMP1 and the Aged-AT-SAMP1 groups compared to that in the Aged-Sed-SAMP1 group (Figure 3A). Additionally, the ratio of CD11c/CD163 and CCR2 mRNA expression increased in the heart, liver, small intestine, brain, aorta, adipose, and TA muscle of the Aged-Sed-SAMP1 group compared to that in the Young-Sed-SAMP1 group, and the ratio was significantly decreased by resistance and aerobic training (Figures 3B,C).

### Chemokine Expression in Tissues

**Figure 4:**
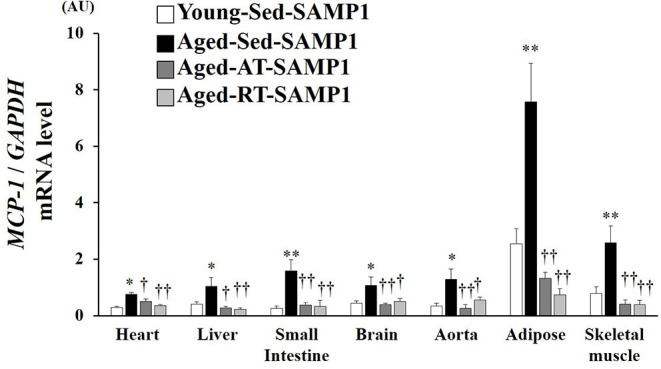
Effects of aging and exercise training on *MCP-1* mRNA expression in inflamed tissues. *GAPDH* mRNA expression was used as an internal control for correcting the mRNA expression of *MCP-1* in the heart, liver, small intestine, brain, aorta, adipose, and skeletal muscle (tibialis anterior muscle) tissues. AU, arbitrary units. The values are expressed as means ± SEM. ^*^p < 0.05 vs. Young-Con-SAMP1; ^**^p < 0.01 vs. Young-Con-SAMP1; ^†^*p* < 0.05 vs. Aged-Sed-SAMP1; ^††^*p* < 0.01 vs. Aged-Sed-SAMP1.

Since macrophage infiltration is regulated by MCP-1, we evaluated MCP-1 mRNA expression levels in inflamed tissues. MCP-1 mRNA expression in the heart, liver, small intestine, brain, aorta, adipose, and TA muscle significantly increased in the Aged-Sed-SAMP1 group compared to that in the Young-Sed-SAMP1 group, and the expression was significantly reduced by aerobic and resistance training (Figure 4).

## Discussion

In this study, we revealed the preventive effects of different exercise programs (including aerobic and resistance training) on chronic inflammation in various tissues in aging mice. In aged SAMP1 mice, the elevation of circulating TNF-α levels, a marker of systemic inflammation, were attenuated by both exercise types, and this effect did not differ between the two types of training. Additionally, an increase in TNF-α and IL-1β mRNA expression levels in the aorta, heart, liver, small intestine, adipose, and skeletal muscle in the aged mice was observed; these elevated expression levels were attenuated by both exercise programs. Interestingly, no significant change in TNF-α and IL-1β mRNA expression levels in the spleen, large intestine, and lung were seen in the two trained mice. Thus, the preventative effects of exercise on age-induced increases in TNF-α and IL-1β mRNA expression levels in local tissues may contribute to the attenuated effect of increase in circulating TNF-α level, as a marker of systemic inflammation. Moreover, even if the exercise manner is different, as with aerobic and resistance training programs, the effects gained from the exercises may be similarly obtained.

Macrophage infiltration and the balance of macrophage polarization are key regulators of chronic inflammation in tissues, and chemokine, such as MCP-1, is required for macrophage infiltration (Oh et al., 2012). This study revealed the differences in the effects of macrophage infiltration as well as polarization in various tissues following resistance and aerobic exercise programs in aged SAMP1 mice. In this study, the increases in expression levels of F4/80 mRNA, a marker of macrophage infiltration in cells, and ratio of CD11c / CD163 and CCR2 mRNA expression, markers of M1/M2 macrophage polarization, with the elevation of MCP-1 mRNA expression level were found in inflamed tissues of aged SAMP1 mice. Moreover, the age-induced changes in macrophage infiltration, M1/M2 macrophage balance, and chemokine in local tissues were restored by both exercise programs. Thus, the effects of exercise on inflamed tissues in aged SAMP1 mice may be involved in the amelioration of macrophage infiltration and M1/M2 macrophage balance; the response of MCP-1 in the local tissues may be involved in these mechanisms.

MCP-1 plays a crucial role in macrophage migration and inflammatory reaction under inflammatory conditions (Mantovani et al., 2004). In the MCP-1 knock-out mice, F4/80 mRNA expression and TNF-α mRNA expression in inflamed tissues of mice was attenuated, which led to the inhibition of chronic inflammation (Chen et al., 2011; van Zoelen et al., 2011; Oh et al., 2012). In this study, resistance and aerobic exercise training attenuated the increased expression levels of F4/80 and TNF-α mRNA in the heart, liver, small intestine, brain, aorta, adipose, and muscle of aged mice as well as the elevated expression levels of MCP-1 mRNA. Hence, the change in MCP-1 levels in various tissues by aging and exercise training may be involved in a regulation of macrophage infiltration in tissues; consequently, this mechanism may play a role in the induction of chronic inflammation in local tissues. Additionally, a previous study has reported that voluntary wheel running for 10–14 weeks reduced the activation of NF-κβ, which induced MCP-1 production in the aorta of aged mice (Lesniewski et al., 2011). Therefore, we hypothesized that the same mechanism may cause the reduction of MCP-1 mRNA expression by two types of exercise training in various tissues.

In this study, we revealed for the first time that there is no difference in the preventive effect of resistance and aerobic training on chronic inflammation with aging. However, the mechanism underlying these immune responses is unclear. Acute exercise-induced secretions of catecholamine may be involved in an underlying mechanism for the inhibition of chronic inflammation. In previous human studies, resistance training has shown to increase blood catecholamine levels (Okamoto et al., 2009), whereas aerobic training increased the expression of β2 adrenergic receptor on leukocytes (Tartibian et al., 2015). Furthermore, our previous study showed that the accelerated secretion of catecholamine by aerobic exercise inhibited the inflammation induced by β2 adrenergic receptor-mediated immune activation (Tanaka et al., 2010). Additionally, catecholamine stimulation in the monocytes of mice enhanced a shift of M2 macrophage polarization via β2 adrenergic receptor (Lamkin et al., 2016). Based on these findings, aerobic and resistance training may attenuate age-associated chronic inflammation through the release of catecholamine.

Furthermore, macrophages are involved in chronic inflammation in tissues. Previous studies have observed that aged mice exhibit a high expression of inflammatory markers and macrophage markers in the liver, adipose, and aorta (Lesniewski et al., 2011; Lumeng et al., 2011; Kim et al., 2016). In addition, aging leads to an increase in M1 macrophage markers and a decrease in M2 macrophage markers in adipose tissue (Toba et al., 2015). However, it is unclear as to the influence of chronic inflammation and macrophage infiltration and polarization into other tissues in aging. We demonstrated that aged SAMP1 mice exhibited an increase in TNF-α, F4/80, and CCR2 mRNA expression and CD11c/CD163 ratio not only in the liver, adipose tissue, and aorta but also in the brain, heart, small intestine, and skeletal muscle. Moreover, the increase in inflammatory markers and macrophage makers was attenuated by the two exercise types in the tissues of aged SAMP1 mice. Our data showed that, in various tissues, age-induced chronic inflammation is involved in macrophage infiltration and polarization. Inhibition of macrophage infiltration and M1 polarization by the two exercise types may attenuate age-induced chronic inflammation in various tissues.

This study examined the mRNA expression levels of several inflammatory parameters, such as TNF-α, IL-1β, IL-6, and MCP-1. Since changes in protein expression levels reflect functional adaptations, future studies should examine not only mRNA levels but also protein expression and concentration levels. Furthermore, we investigated the mRNA expression levels of F4/80, CD11c, and CD206, the molecular markers of macrophage infiltration and M1 and M2 macrophages, respectively. However, F4/80 mRNA expression in the adipose tissue of obese mice reflected macrophage activation rather than infiltration (Bassaganya-Riera et al., 2009). In addition, CD11c was expressed not only in macrophages, but also in dendritic cells (Grolleau-Julius et al., 2006). Though we measured mRNA expression of CCR2, as an inflammatory macrophage marker, changes in F4/80 mRNA expression and the ratio of CD11c/CD206 mRNA expression alone may not reflect macrophage infiltration and polarization of all tissues. Therefore, further studies are required to determine the extent of macrophage infiltration and the phenotypic switch of macrophage polarization using histological analysis. Finally, this study examined the preventive effect of exercise on age-induced chronic inflammation using SAMP1 mice, a senescence-accelerated mouse model. Future studies need to examine the effect of resistance and aerobic training on age-induced chronic inflammation using C57Bl/6 mice.

In summary, resistance and aerobic exercise training might ameliorate gene expression profiles for macrophage infiltration and polarization in various tissues of aged SAMP1 mice, and these alterations might be involved in the prevention of age-related tissue chronic inflammation and lead to a reduction of the increase in circulating TNF-α level, as a marker of systemic inflammation, in aged SAMP1 mice.

## Data Availability Statement

All datasets generated for this study are included in the manuscript/supplementary files.

## Ethics Statement

Ethical approval for this study was obtained from the Committee on Animal Care at Ritsumeikan University (BKC2017-024).

## Author Contributions

MI, MU, and NHo designed research and wrote the paper. MI, MU, NHo, NHa, SF, EO, and HY performed research. MU and NHo analyzed data.

### Conflict of Interest

The authors declare that the research was conducted in the absence of any commercial or financial relationships that could be construed as a potential conflict of interest.
